# The Role of Relevance in Shaping Perceptions of Sleep Hygiene Games Among University Students: Mixed Methods Study

**DOI:** 10.2196/64063

**Published:** 2024-10-08

**Authors:** Zilu Liang, Edward Melcer, Kingkarn Khotchasing, Samantha Chen, Daeun Hwang, Nhung Huyen Hoang

**Affiliations:** 1 Ubiquitous and Personal Computing Lab Kyoto University of Advanced Science Kyoto Japan; 2 School of Computer Science Carleton University Ottawa, ON Canada; 3 University of California, Santa Cruz Santa Cruz, CA United States

**Keywords:** serious games, sleep hygiene, sleep technologies, co-design, relevance, self-determination theory, digital health, persuasive technology, behavior change

## Abstract

**Background:**

Sleep games are an emerging topic in the realm of serious health game research. However, designing features that are both enjoyable and effective at engaging users, particularly university students, to develop healthy sleep habits remains a challenge.

**Objective:**

This study aims to investigate user preferences for 3 sleep game prototypes, that is, Hero’s Sleep Journey, Sleep Tamagotchi, and Sleepland, and to explore their popularity and perceived utility in promoting sleep health.

**Methods:**

A mixed methods approach was used in this study. Quantitative and qualitative data were collected through a co-design workshop involving 47 university students. Participants were presented with storyboard cards of game features and were asked to provide an overall rating on each game, as well as ratings for individual features. They were also encouraged to provide free-form comments on the features and suggest improvements. In addition, participants were asked to express their preferences among the 3 games regarding which game they would most like to play and which one they found most useful for promoting sleep health.

**Results:**

Surprisingly, while Hero’s Sleep Journey was the most popular choice among participants, Sleep Tamagotchi was perceived as the most beneficial for improving sleep health. Relevance emerged as an overarching theme in the qualitative data analysis, with 3 interconnected dimensions: psychological relevance to users’ personal lives, logical relevance to sleep health, and situational relevance to users’ circumstantial context. We discussed how the 3 dimensions of relevance address the autonomy and relatedness constructs outlined in the self-determination theory and proposed 3 design recommendations.

**Conclusions:**

Our serious sleep game prototypes demonstrated the potential to engage university students to develop healthy sleep hygiene. Future sleep game designs should aim to create a sense of relevance to users’ personal lives, sleep health goals, and situational contexts. Rather than a one-size-fits-all approach, it is essential to develop a wide range of game genres and features to cater to diverse users. Aligning game features with sleep health goals and educating users on the design rationale through sleep knowledge are also important aspects. Furthermore, allowing users to customize their game experience and manage technology boundaries is necessary to nurture a sense of control and autonomy in the process of forming good sleep hygiene.

## Introduction

### Background

Sleep plays an important role in sustaining physical and mental health [1]. Not having enough good sleep is associated with an increased risk of cardiovascular diseases, metabolic disorders, temporary and permanent cognitive impairment, and mental health issues [2,3]. However, attaining adequate sleep is often challenging for many university students, and poor sleep quality is frequently associated with poor sleep hygiene in this population [4-7]. Sleep hygiene encompasses 4 interconnected dimensions: establishing a regular sleep-wake cycle, creating a nighttime routine, optimizing the sleep environment, and cultivating healthy daily habits [8]. Sleep research has demonstrated that behavior changes targeting sleep hygiene positively impact sleep health among clinical populations [8,9], indicating the potential application of behavior change technology for promoting healthy sleep behavior. Despite this potential, the development of such technologies in the domain of sleep health has generally been limited in focus and scope [10].

There have been previous attempts to incorporate playful elements into improving sleep hygiene to make the process more engaging and effective [11]. These efforts can be broadly categorized into tangible approaches and mobile apps. Tangible approaches use physical tools or materials, such as worksheets [12], tactile game boards [13], and aroma tokens [14]. In addition, various alarm clocks have been introduced with designs to awaken users in effective and creative ways. Examples include Ruggie (Windustries Limited), which requires users to step on it; Gun O’Clock (Bandai Namco), which necessitates shooting a target; and Clocky (CLOCKY, LLC) and the Helicopter Alarm Clock (Toyo Trading), which must be chased to deactivate. These approaches challenge users by incorporating active tasks to turn off the alarm.

With respect to mobile apps, many attempts have been made to develop various sleep games. Some focus on relaxation by integrating peaceful and calm elements within the game design (eg, Harmony [Logitech Europe SA], SpinTree 3D [Tabasco Games], and Sheep Sleep [Superpea Ltd]), while others guide users through relaxation techniques such as breathing exercises (eg, Loona [KEYi Technology Co, Ltd] and Mindllama Breathe Sleep better [Llama Luna Apps]). Certain games promote mindfulness regarding bedtime routines [15], screentime before bed [16], and daily habits related to sleep [14] or incentivize the maintenance of healthy bedtime habits (eg, Sleep Town [Seekrtech] and Pokémon Sleep [The Pokémon Company]). Despite this variety, most of these apps function as alarm clocks, offering playful methods to wake users up and regulate their sleep patterns. They typically present specific missions (eg, Early Bird Alarm Clock [ONEYEAR], Sleep as Android: Smart alarm [Urbandroid Team], AlarmClock Xtreme [Agilesoft Resource], ChallengeAlarm [Nicole Ocean], Alarmy [Delight Room Co, Ltd], and TurboAlarm [Francisco Javier Castaño Gómez]) or incorporate minigames, such as simple shooting games or puzzles, requiring players to achieve certain goals to deactivate the alarm (eg, AlarmMon [Malang Studio Co Ltd], Vmons [Vmons App], Wakey [Kanetik], AlarmBuddy [Alarm Buddy], and Unicorn Alarm [NETIGEN Apps]).

While there are numerous games and gamified apps to enhance sleep hygiene, there remains significant room for improvement. First, as noted earlier, existing solutions are primarily designed for mobile apps or custom tangible artifacts. At this point, smartwatches offer unique advantages over other types of technology, such as reduced blue light exposure, timely interventions, and the capacity to track different health indicators [17,18] and sleep metrics [19], with reasonable accuracy [20]. Nevertheless, surprisingly little work has explored the potential of smartwatch games for promoting sleep hygiene.

Second, current sleep games and gamification systems primarily target the nighttime routine and, to a lesser degree, the regularity of sleep-wake cycles and sleep environment optimization. Only 1 study has explored the design space surrounding daily activities, particularly caffeine intake, for sleep hygiene improvement [14]. Targeting daytime activities that promote better sleep represents a vast design space, including strategies, such as receiving early morning sunlight exposure, having meals early, and avoiding late naps or caffeine consumption close to bedtime [21-25]. Furthermore, it is still unclear how incorporating daily activities to improve sleep health will be received by users of sleep hygiene technologies.

Finally, most current services aim to improve sleep quality through gamified apps rather than actual games that provide a fully playful and immersive experience. The potential of games for behavior changes and persuasive technologies is well documented [26] but surprisingly underexplored in the domain of sleep health.

### This Study

This work seeks to explore how smartwatch-based sleep health games are perceived by potential users and could in turn be used as effective tools for improving sleep hygiene. We achieve this by tapping into the unexplored dimensions of sleep hygiene games using a participatory design approach, aiming to provide insights and design implications that contribute to the future development of serious sleep hygiene games.

## Methods

### Conceptualization and Design of Smartwatch Sleep Games

#### Overview

**Figure 1:**
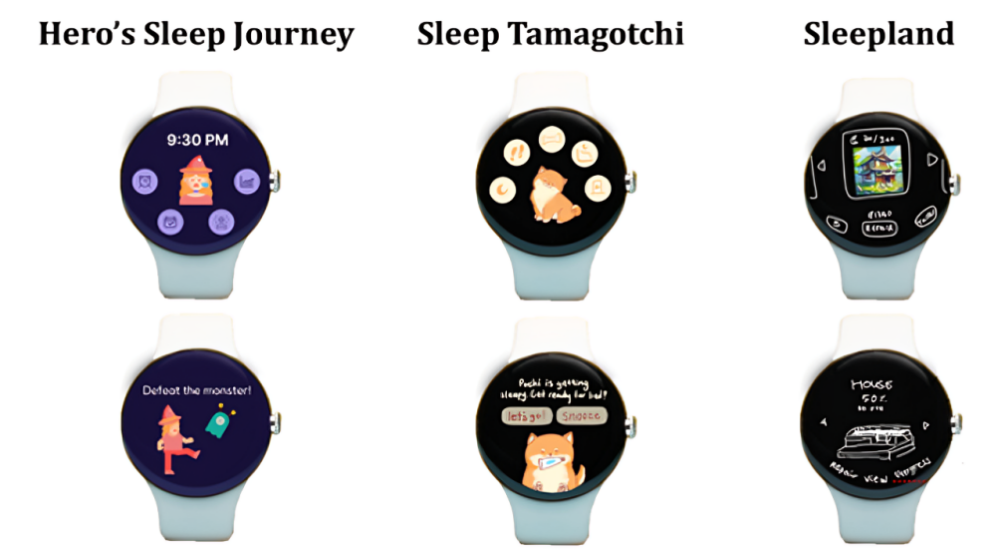
Prototype mockups of the 3 smartwatch sleep games.

In prior work, we adopted a co-design approach that gathered a cohort of university students to participate in the brainstorming and design of sleep health technologies targeted at addressing the sleep health needs of this population more accurately [4]. A total of 51 university students participated in 3 co-design workshops. Thematic analysis of the qualitative data generated 9 themes, including health tracking, sleep environment optimization, sleep literacy, community, recommendation, gamification, generative artificial intelligence, materializing sleep with learning, and personalization. For more details on the workshop and resulting initial design insights, refer to the study by Liang et al [4]. Building on these design insights and evidence-based sleep health knowledge, we developed 3 low-fidelity prototypes of smartwatch sleep games that addressed various themes identified as important by the participants of the initial workshop (Figure 1). The game features for each prototype and their designs are described in more detail in the subsequent sections.

#### Hero’s Sleep Journey: Power Up Hero With Your Sleep to Fight Monsters

Hero’s Sleep Journey (HSJ) focuses on leveraging the powerful motivational effects of role playing and avatars [27-29]. It features a hero avatar as the main visual theme and is centered on adventure and mission completion. Users play as a hero who grows stronger through good sleep hygiene and fights monsters to protect their village. Some of the core game features are summarized in Textbox 1.

**Summary of core game features of Hero’s Sleep Journey.**

**Core game features of Hero’s Sleep Journey**

- Regain health points through sleep: players restore their hero’s vitality by ensuring adequate sleep, with an emphasis on sleep duration and quality.
- Fight monsters through healthy daily habits: players fight monsters that attack the village by performing various healthy daily habits, such as exercising, walking, stretching, and so forth. Successfully defeating monsters rewards players with in-game currency, which can be used to hire additional heroes or change avatars, as well as buy accessories and weapons that can further improve their hero’s abilities.
- Complete daily sleep hygiene challenges: players can complete a small number of special challenges each day to gain unique rewards for their hero, such as extra currency, exclusive weapons or accessories not available in the shop, and stat increases that can further improve their hero’s abilities. Examples of special challenges include but are not limited to walking a set amount of distance, taking a certain number of steps in an hour, or reaching a specified heart rate.

#### Sleep Tamagotchi: Raise Virtual Pets With Your Sleep

Sleep Tamagotchi (ST) leverages the charm of a virtual pet to engage players in improving their sleep hygiene [30,31]. The central theme involves a cute digital pet that guides users in developing and maintaining healthy sleeping habits. Some of the core game features are summarized in Textbox 2.

**Summary of core game features of Sleep Tamagotchi.**

**Core game features of Sleep Tamagotchi**

- Complete daily routines with pet: players can complete various daily activities with their pet, such as stretching, walking, and exercising at appropriate times of the day. These activities are proven to benefit night sleep according to sleep science studies [6,7].
- Prompting healthy sleep practices: players will be prompted with notifications and in-game animations of their pet performing desired activities at appropriate times of day or night (eg, walking, stretching, taking a bath, getting ready for bed, and sleeping).
- Customization rewards for healthy sleep practices: players earn rewards and gifts for their pet through sleeping well, maintaining a consistent sleep schedule, and forming good sleeping habits.

#### Sleepland: Building Village With Your Sleep

Sleepland focuses on using the motivational power of construction games, that is, games where players combine or configure objects to organize and build things [32]. Specifically, it features building construction as its main visual theme, allowing players to create and develop their own land or town. Users are tasked with the challenge of restoring a Japanese village using their *sleep power*, which is accumulated through healthy sleep hygiene activities. Some of the core game features are summarized in Textbox 3.

**Summary of core game features of Sleepland.**

**Core game features of Sleep Tamagotchi**

- Engaging game narrative: A guardian spirit visits players and asks for the player’s help in fixing an old countryside village so that they can have a beautiful place to watch over and protect again. The player has daily interactions with this guardian spirit and slowly learns about the village, its people, and its history over time as they progress through the game.
- Healthy sleep hygiene rewards: When players sleep, they can meet goals and reinforce healthy behaviors to gain points. Meeting goals for the first time, such as sleeping earlier or reducing screen time, will allow them to repair the village and expand its boundaries. In addition, maintaining existing healthy sleep behaviors will reward the player with in-game currency, which can be used to build within the village and customize its look.
- Repair the village: The repair stage involves fixing habits that users want to change, such as waking up earlier, sleeping earlier, reducing screen time, or drinking more water for the first time. Meeting these goals allows users to repair places and items in the village.
- Build within the village: Players can build new habits by repeating healthy behavior over time. As players build up these new habits, they receive in-game currency that allows them to build new things within the village to customize its look, layout, and feel.

### Co-Design Workshop

#### Participants

We distributed flyers and posters around the campus of the Kyoto University of Advanced Science (KUAS). A total of 45 participants (self-identified as female participants: n=7, 16%; and self-identified as male participants: n=38, 84%) attended the workshop. The age of the participants ranged between 18 and 35 years. In total, 9 (20%) participants had prior experience using a smartwatch or wristband for self-tracking. All participants were undergraduate or graduate students enrolled in the Faculty of Engineering at KUAS during the time of the workshop.

#### Workshop Flow

**Figure 2:**
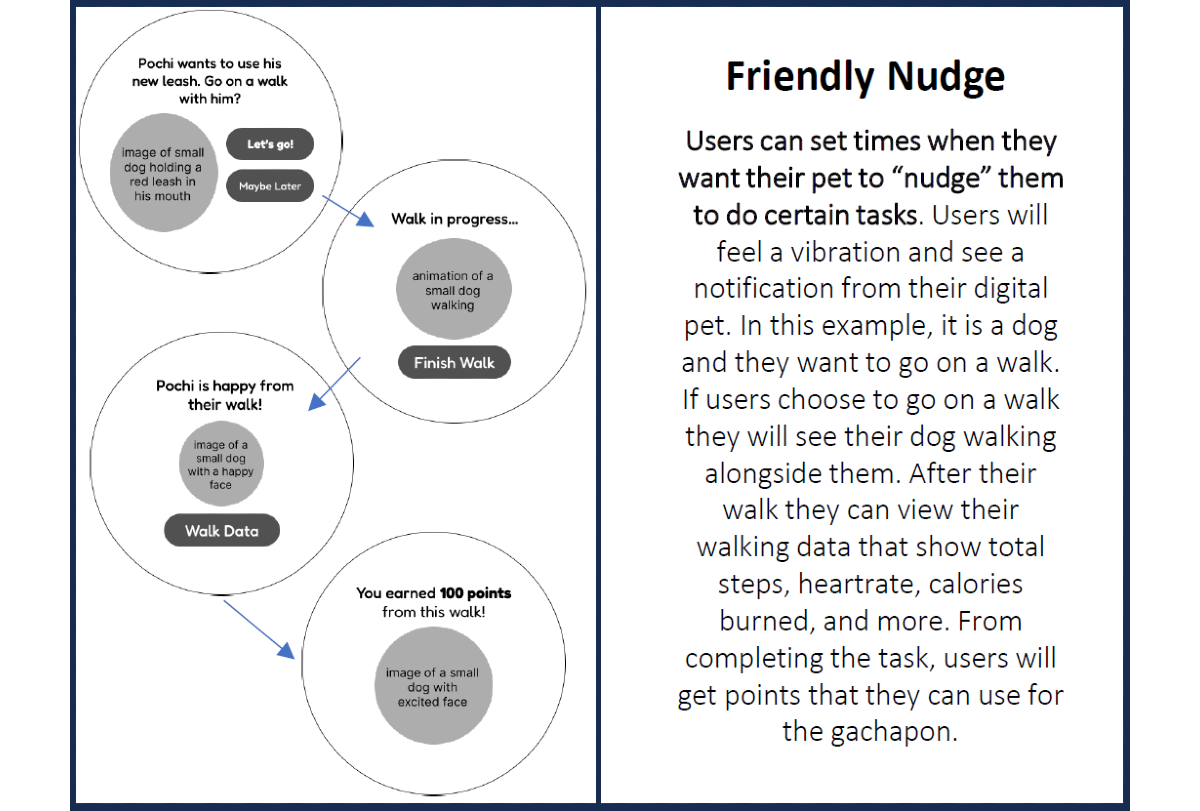
Storyboard of the friendly nudge feature in Sleep Tamagotchi.

**Figure 3:**
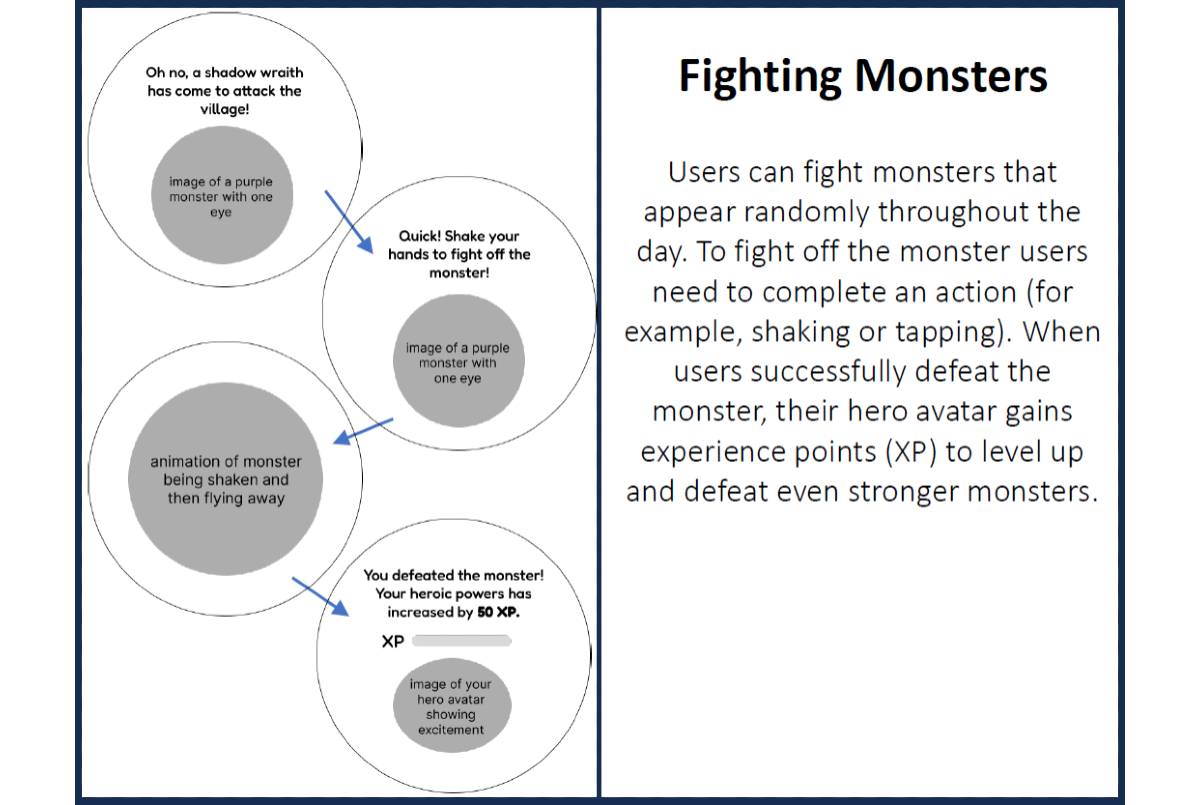
Storyboard of the fighting monster feature in Hero’s Sleep Journey.

Before beginning the main activities of the workshop, we provided an introduction to the workshop goal as well as background information on serious games, wearable sleep-tracking technologies, and sleep hygiene. Participants were given an icebreaker activity to encourage collaboration and discussion among one another. Following the icebreaker, participants were briefed on the main findings from the initial workshop and given background information about the sleep game. They were then presented with storyboard cards depicting features from all 3 prototypes. In total, 2 examples of these storyboard cards are shown in Figures 2 and 3. Each card included 4 questions on the backside: “How useful is this feature for improving sleep?”; “Would you use this feature?”; “Could anything be improved?”; and “Free comments.” The first 2 questions required participants to rate the feature on a Likert scale ranging from 1 to 4, with higher ratings indicating a more positive appraisal of the feature. The last 2 questions are open-ended. Participants formed groups of 4 to 6 people to evaluate each feature of the 3 prototypes and were encouraged to openly share their feedback within their groups. Once all features were evaluated, the cards were collected.

At the conclusion of the workshop, participants completed the 5-factor player traits questionnaire [33], a validated instrument with 25 items, administered in paper format. This questionnaire assessed participants’ preferences for different game elements and playing styles. Using this tool provided a deeper insight into the participants’ inclinations toward each sleep game. Alongside the questionnaire, participants were also surveyed about the amount of time they spent playing games per week as well as their preferences among the 3 games regarding which one they found most useful for promoting sleep health and which game they would most like to play.

#### Data Analysis

This study used a mixed methods approach, resulting in a comprehensive collection of both quantitative and qualitative data from the workshop. Initially, all data were collected on paper and subsequently digitalized into spreadsheets for analysis. For the quantitative data analysis, histograms were created to illustrate participants’ ratings of each game’s overall appraisal and individual features. The qualitative data, consisting of participants’ free-form comments, were analyzed using thematic analysis. In total, 3 authors independently conducted the coding, and discrepancies were resolved through discussion. Instead of using a predefined coding schema, codes were inductive and identified iteratively by repeatedly reviewing the comments. These codes were then grouped into 8 themes, which will be detailed in the *Qualitative Findings* section.

### Ethical Considerations

The study was approved by the ethics review board of Kyoto University of Advanced Science (23E04). Participants signed informed consent forms before the start of the workshop and received an Amazon gift card (approximately US $20) upon the completion of the workshop. Data were anonymized to protect the privacy of the participants.

## Results

### Participants

Understanding player traits is essential for designers to create games that resonate with their target audience. We received 96% (43/45) of the responses to the 5-factor player traits questionnaire. Nearly half (21/43, 49%) of the respondents primarily demonstrate the esthetics trait, highlighting their appreciation for a game’s visual and auditory elements, such as graphics, sound, and art style. The second most common trait is the narrative trait (11/43, 26%), revealing their preference for complex storylines within games. In addition, 21% (9/43) demonstrated dominant social traits, reflecting their preference for social interaction during gameplay and their desire to feel connected to other players. As for game-playing hours, most respondents (31/43, 72%) reported spending a modest amount of time playing games, typically between 1 and 10 hours per week, which aligns with the global average of around 8.5 hours per week [34]. In addition, 19% (8/43) spent 11-20 hours per week, while only 7% (3/43) spent more than 21 hours per week on gaming.

### Quantitative Findings

We received responses from 96% (43/45) of participants regarding their preferences for the 3 sleep game prototypes. Our results revealed a notable discrepancy between the popularity and perceived usefulness of sleep games. When asked which game prototype they would most like to use, 56% (24/43) of the respondents selected HSJ, 30% (13/43) chose ST, and only 14% (6/43) opted for Sleepland. By contrast, when asked which game prototype they found most helpful for improving sleep, 67% (29/43) identified ST, 23% (10/43) picked HSJ, and only 9% (4/43) selected Sleepland.

For the perceived utility of individual features, we received ratings and feedback from 91% (41/45) of participants for both Sleepland and HSJ and 84% (38/45) of participants for ST. Table 1 shows the portion of respondents who answered “yes” to the question “Would you use this feature?” The most popular features across 3 sleep games are sleep reports, sleep rewards, and daily challenges, which have a clear and direct relation to changing sleep hygiene behaviors and providing concise feedback on that progress. In contrast, the least popular features are fighting monsters and hero selection in HSJ and sleep education in ST. These features either obfuscated the focus on sleep hygiene or required extraneous amounts of effort to process.

**Figure 4:**
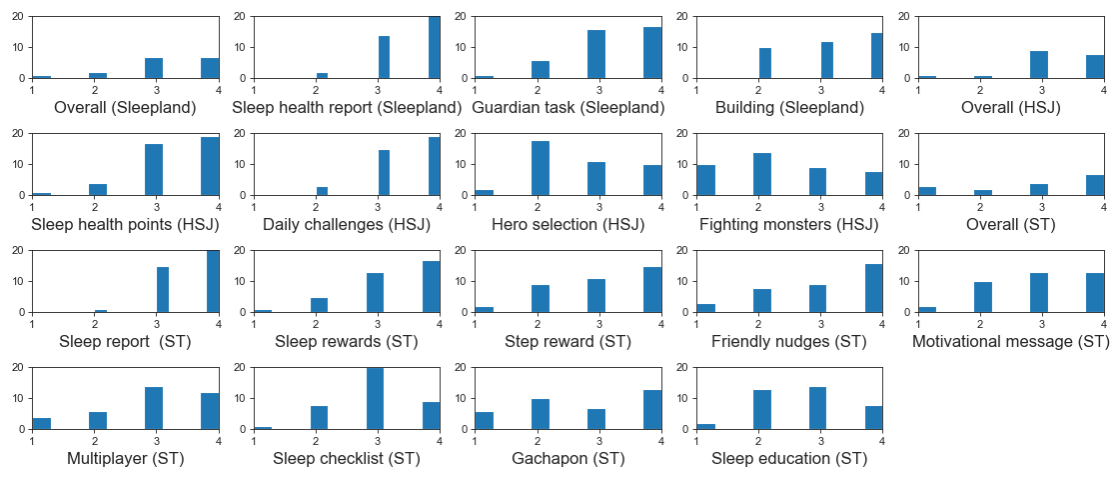
Histogram of participants’ ratings to the question “How useful is this feature for improving sleep health?” (1=not useful and 4=very useful). HSJ: Hero’s Sleep Journey; ST: Sleep Tamagotchi.

A similar tendency was observed in the histograms of respondents’ ratings for each game prototype and individual feature, as illustrated in Figure 4. Consistent with participants’ willingness to use the features, the hero selection and fighting monster features of HSJ as well as the sleep checklist and sleep education features of ST received lower ratings from participants. In contrast, the sleep health report and Guardian’s task features of Sleepland, the sleep health points and daily challenges features of HSJ, and the sleep report and sleep rewards features of ST received higher ratings, indicating greater appreciation for these features. Meanwhile, features such as Gachapon and motivational nudges of ST exhibited more variance in their scores.

**Table 1:** The proportion of respondents who answered “yes” to the question “Would you use this feature?”

Game and individual feature			Respondents, n (%)
**Sleep Tamagotchi** **(n=38)**			
	Sleep report	33 (87)	
	Sleep rewards	32 (84)	
	Motivational message	27 (71)	
	Sleep checklist	27 (71)	
	Step reward	26 (68)	
	Friendly nudges	26 (68)	
	Multiplayer	25 (66)	
	Gachapon	24 (63)	
	Sleep education	19 (50)	
**Hero’s Sleep Journey** **(n=41)**			
	Sleep health points	37 (90)	
	Daily challenges	34 (83)	
	Hero selection	26 (63)	
	Fighting monsters	18 (44)	
**Sleepland** **(n=41)**			
	Sleep health report	36 (88)	
	Guardian task	33 (80)	
	Building	31 (76)	

### Qualitative Findings

Thematic analysis identified 8 themes, with 2 that arose from participants’ suggested improvements for each game or feature.

#### Game Genre Impacts the Popularity of Sleep Games

Our observations suggest that the genre of the game influences participants’ perceptions of its features. Many participants who indicated their preference for HSJ were originally fans of role-playing games (RPGs), drawn to the fantasy environment embodied by the theme and storyline of the HSJ game. For instance, the participants said the following:

(The HSJ game) has a clear energy and having to sleep to fight is pretty cool.

P24

It’s a good idea to have a role-playing game. Having a main hero character has always been the ‘apple of the eye’ for people in movies and games. So having a hero against a villain is a good idea for games.

P35

Similarly, participants who favored Sleepland were originally fond of building games, while those who chose ST felt connected to animals and pets. They said the following:

I love world-building games where you can create your own little corner of peace!

P4

I like gathering resources and using them to build something up, and this would be a fun way to get points and work towards a satisfying village.

P10

I really enjoy games related to pets that we have to take care of or fight for.

P23

#### Different Preferences Between Competitive and Noncompetitive Players

Another primary factor influencing participants’ preferences for different sleep game prototypes was their orientation toward competitive or noncompetitive gameplay. The HSJ game distinguished itself as the most interactive game among the 3, featuring level-up mechanics and customization that appealed to competitive players. P34 noted the following:

The hero character is very interesting with the upgrade potential. I could make my own customizable character different from my friends.

P34

In addition, a few participants found HSJ useful for improving sleep due to its motivating and engaging nature. In total, 4 participants who voted for HSJ believed that the daily challenges would motivate them to “be more mindful of sleep health” and they would “try to sleep well so that the hero can win.” P13 mentioned that “progress can be made much faster (in HSJ) compared to the other two (games) due to the element of competition.”

Conversely, not all participants identified as competitive players. Some preferred Sleepland and ST precisely for their passive nature. Compared with HSJ, Sleepland was considered less complicated because, as P3 said, “the tasks can be automated, and it takes minimum input from the users.” The pet theme of ST was also perceived as more lighthearted compared with the other 2 games, as P5 noted, “I don’t think I would be a competitive sleeper, which is the requirement for Hero’s Sleep Journey.” Unlike HSJ, which excites players, ST causes less stress as, according to P5, “it seems like a fun app in an easygoing manner. Not too competitive. And feels like a very gently encouraging app.”

#### Emotional Impact of Sleep Games

Participant feedback highlights the emotional impact of sleep games. Interacting with cute pets in ST seems to create an affection effect that reinforces positive behavior change by fostering a sense of companionship and mirroring behavior. Participants reported feeling connected to virtual pets, with some expressing willingness to “do anything for a cute virtual pet, even sleeping early” or “try to complete any tasks that a virtual pet gives me.” Furthermore, the friendly nudge feature in ST was praised for its companionship effect, potentially enhancing users’ motivation. P5 commented as follows:

It might be a bit boring to do walks or exercises on your own, so it would be nice to have some encouragement from a virtual pet.

P5

Virtual pets may also have a mirroring effect, as stated by P40:

When you are taking care of the animals routine, you can have a perceptive of your own routine.

P40

A similar effect was also noted in the HSJ game. One participant associated the healing during sleep with the healing process of the hero character, thus considering an RPG the most suited for sleep health intervention.

Nevertheless, we also observed potential negative emotions that sleep games may elicit, particularly in relation to reward features. While rewards can serve as external motivation for some participants, others expressed concerns that earning health points or completing tasks in HSJ may induce unnecessary stress. In addition, rewards based on sleep quality may inadvertently trigger rumination, since factors like the amount of deep sleep are often beyond an individual’s control.

#### Alignment With Sleep Health Enhances Perceived Utility of Game Features

Surprisingly, the most popular game is not necessarily perceived as the most useful. While HSJ seemed to be the most popular, ST was perceived as the most useful. The primary reason for ST’s perceived utility lies in its features, which are more focused on sleep health and less distractive from the sleep goal.

More than one-third (13/38, 34%) of the participants considered the ST features highly pertinent to sleep health, stating that ST “has the most sleep and helpful driven features out of the three” (P12). They acknowledged that “there are lots of features that could help me improve my sleep health even if the features or tasks might not be interesting” (P22). Sleep report and sleep education were 2 features that participants highly appraised for their potential positive impact on users’ awareness of sleep health. They considered the sleep education contents useful and informative, and the sleep reports would allow users to “look back in the long run” to “analyze sleep patterns” so that “users can be in touch with this for a longer period without getting bored” (P43). These features help users stay focused on improving sleep health without overengaging with features that are not critical for sleep health. As P30 stated, “the other games will make you get distracted and prioritize your game more than your sleep.”

Similarly, due to the perceived direct link to sleep, the sleep reward features in all 3 prototypes, which manifested differently as a building feature in Sleepland, a sleep health points feature in HSJ, and a sleep reward feature in ST, were highly praised by participants. In contrast, the step reward feature in ST, while also a form of reward, garnered less interest. Given the unclear relationship between step count and sleep quality improvement, participants were less inclined to engage with this feature.

#### Game Features Distracting From Sleep Health

We observed that some interactive features, despite being fun and engaging, were considered distracting from the goal of improving sleep health. Participants noted that the diversity of tasks in HSJ is likely to keep users engaged. P1 stated the following:

The range of possible tasks is the most diverse in Hero’s sleep journey, so it is most likely to keep me engaged the longest.

P1

However, participants also acknowledged that these interactive features are not necessarily tied to improvement in sleep quality, just as one participant mentioned:

It looks the most fun to me. It also feels like the most interactive one. Although I don’t feel like it would help improve my sleep like the other two.

P12

Similar complaints were noted about Sleepland by P12, “it (Sleepland) feels more like Clash of Clans than a sleep improving app.”

#### Features Influencing Perceived Autonomy

Participants’ preferences for game features were influenced by their perception of autonomy while engaging with those features. They highly valued the flexibility of tasks in Sleepland and ST, emphasizing the importance of being able to “choose their tasks” and “do them at any time” to avoid interference with their work or class schedule. In contrast, the hero selection feature in HSJ received criticism for requiring frequent user choices. Excessive customization imposed a sense of forced action that compromises autonomy. Similarly, the sleep checklist feature imposes a sense of forced action. Participants stated that they would be “too tired at the end of the day to check the list” (P12) and “people have different strategies in winding down for sleep” (P22).

The daily challenge features, including the friendly nudge feature in ST, a daily challenge feature in HSJ, and the Guardian’s task feature in Sleepland, may also compromise autonomy. For example, participants found that the 10,000-step goal was too laborious and challenging to achieve, preferring apps that do not dictate their actions. P45 preferred to do things by herself, and she did not “need the app to tell me what to do.” In addition, concerns were raised regarding the potential frequent notifications resulting from the daily challenge features. Participants expressed worries that these notifications could be disruptive and cause annoyance rather than motivation.

### Suggested Improvements

#### Overview

Participants offered valuable feedback for improving the sleep games, as summarized in Textbox 4. The suggestions largely fall into 2 categories: enhancing the game experience and aligning the game with sleep health goals and outcomes. Suggestions for improvement included customization options, diverse reward structures, and integration of community features. In addition, participants emphasized the importance of aligning game features with sleep health goals, such as discouraging oversleeping and providing personalized sleep recommendations.

**Summary of participants’ suggestions for improvement.**

**Suggestions for enhancing the game experience**

- Avoid excessive notifications
- customization (game characters, daily challenge list, and goals)
- personalized reward (bonus rewards for streaks and higher rewards for people with a worse sleep baseline)
- community and social features
- support multisensory interaction (voice input and vibration)

**Suggestions for better alignment with sleep health**

- Provide personalized sleep recommendations
- align daily challenges and rewards with the day-night cycle
- discourage oversleeping

#### Suggestions for Enhancing Game Experience

Participants highlighted the need to maintain personal space and avoid excessive notifications, which can become intrusive and bothersome. Allowing users to customize their notification preferences, including whether and when to receive notifications, was considered essential. Customization in other areas of the game was also valued. Along the same line, daily challenges should be tailored to match a user’s capability and daily routine. As P1 stated, “a nice middle ground must be found regarding the difficulty of the daily challenges so as to not demotivate the player.” Diversifying game resources, such as introducing various hero and pet types and various forms of in-game currency such as gold and mana, was suggested to enhance the gaming experience. Participants also proposed the idea of assigning different heroes with unique skills that tackle a set of “daily challenges tailored to specific aspects of sleep hygiene” (P27). For example, 1 hero could “focus on exercise for better sleep,” while another could “focus on sleep preparation” (P27).

Participants highly valued the sleep reward features in all 3 games but suggested that rewards should not follow a linear structure. They proposed awarding bonus points for maintaining streaks and offering higher rewards to those with a low sleep baseline as strategies to encourage consistent, healthy habits and recognize users’ progress. In addition, some participants expressed interest in incorporating penalties for not adhering to good sleep schedules or neglecting daily challenges. They also appreciated the concept of progressive difficulty in the game, with the challenge levels increasing as users advanced. This approach may help maintain engagement and interest while fostering a sense of accomplishment over time.

They also advocated for more community and social features in the games, such as fostering collaboration, competition, team play, and enabling users to share or trade items with other players. For instance, 1 participant suggested allowing users to “visit and interact with other users’ villages” in the Sleepland game.

Furthermore, participants valued the use of multisensory interaction. They proposed the integration of pet sounds, ringtones, and vibration patterns as effective ways to boost user engagement and immersion in the ST game.

#### Suggestions for Better Alignment With Sleep Health

Participants also offered a variety of suggestions to align game features with sleep health, that is, using more endogenous designs [35,36]. These proposals aim to integrate gaming experiences with healthy sleep habits and encourage users to develop better sleep practices grounded in scientific evidence in sleep research.

While recognizing the value of checking sleep statistics, participants emphasized the importance of providing personalized recommendations based on users’ current sleep patterns and predicted future sleep quality. Several participants emphasized the importance of aligning daily challenges and rewards with the day-night cycle. For the Sleepland game, P35 suggested implementing a timeline for tasks, such as “sleeping only at night to gain points and exercise in the morning to earn points.” P3 recommended that “sleeping during specific hours of the day should allow the hero to heal faster.” To minimize distractions from the app during sleep hours, P10 proposed that “if the village is viewed during designated sleep time” in the Sleepland game, “some repairs should be undone.”

Oversleeping appears to be a common issue among university students. Participants underscored the importance of discouraging oversleeping, even suggesting penalties for doing so. As P35 noted, “sleeping too much (more than 7-8 hours) should result in point deduction.”

By contrast, a few participants expressed concerns about rewarding players based on sleep quality, arguing that rewards should instead focus on sleep habits and discipline, such as maintaining a consistent sleep schedule. They noted that sleep quality can be beyond a person’s control, whereas habits and discipline are more manageable.

## Discussion

### Principal Findings

All 3 sleep games incorporate various persuasive strategies, including self-monitoring, reminders, and rewards, addressing the themes that emerged in our initial co-design workshop [4]. However, not all games or their individual features were perceived as equally useful or engaging. Our findings underscore the pivotal role of the relevance of game features in participants’ perceptions of the utility of sleep games. Relevance can be broadly defined as something “important to the matter at hand.” The search for relevance is a fundamental aspect of human cognition, and things are relevant to an individual when they have a positive cognitive effect [37]. The relevance of game experience has been recently explored in [38]. In the field of serious games, the importance of relevance has been studied in learning games [39,40]. Previous research has found that relevance influences motivation, adherence, and initial technology adoption. While the importance of relevance has been somewhat recognized in health games [41], no study has thoroughly explored this topic.

Through our analysis, 3 interconnected aspects of relevance emerged: relevance to users’ personal experience, relevance to sleep health, and relevance to users’ situational context. We mapped these 3 dimensions of relevance in sleep games to different types of relevance identified in the literature: psychological relevance, logical relevance, and situational relevance [42]. Through the lens of self-determination theory [43,44], we discuss how these dimensions of sleep game relevance can fulfill users’ psychological needs, particularly autonomy and relatedness, thereby promoting healthy sleep behavior change.

### Relevance of Sleep Game to Players’ Personal Experience and Preferences

#### Overview

Participants’ inclination to engage with sleep games is heavily influenced by their prior game experience and personal preferences, highlighting the importance of creating psychological relevance in the design of serious sleep games. Our findings indicate that participants preferred sleep games that aligned with their favorite game genre. For example, those fond of RPGs favored HSJ, while others attracted to pet-related themes were more inclined toward ST. Participants’ preferences for sleep games were also shaped by their orientation toward competitive or noncompetitive gameplay: competitive players preferred HSJ, and noncompetitive players leaned toward ST and Sleepland.

Several participants emphasized their sense of connection to the game characters as a main reason for their willingness to engage with the game, further highlighting the importance of sleep hygiene games to create psychological relevance. In HSJ, for example, the hero healing feature serves as a metaphor for the restorative effects of sleep, framing the player’s actions as instrumental in restoring the hero’s vitality. Similarly, users care for virtual pets in the ST game, prompting them to reflect on their own schedules as they maintain a regular schedule for the virtual pet. This creates a digital twin effect [4], aligning with the strategy of similarity used in other health-interventional games [26]. The sense of psychological relevance reduces psychological barriers and enhances the sense of autonomy for behavior change. In contrast, the guardian’s task feature in Sleepland, which involves repairing damaged villages, did not seem to foster the same association with the healing benefits of sleep for humans, possibly due to a lack of similarity and relevance between repairing a lifeless object and human recovery. The importance of establishing an emotional connection in serious games has been previously highlighted in [45-47]. Our findings contribute further evidence within the context of sleep health. Game design elements that foster psychological relevance to players’ prior game experiences and personal lives, particularly those that elicit feelings of autonomy and relatedness, may be crucial to the initial adoption of sleep game technologies.

#### Design Recommendation 1: Diversify Game Genres and Create Relatable Characters

Sleep games need not adhere to a one-size-fits-all design. Instead, offering a variety of game genres allows users to choose those that resonate most with their preferences and personal experiences, thereby transforming the external demand for healthy sleep behavior change into a more self-determined, personally endorsed, and volitionally initiated process. Furthermore, creating game characters that users feel connected to will enhance perceptions of self-relevance and promote a sense of relatedness [48-50]. This, in turn, boosts intrinsic motivation to engage with the game and healthy behavior change.

### Relevance of Sleep Game Features to Sleep Health

#### Overview

The second dimension of relevance for sleep games revolves around whether the game features logically contribute to improving sleep. As a form of serious game, sleep games use game design elements to influence players’ behavior toward better sleep health. Aligning game design with the serious context poses a well-acknowledged challenge in serious game research [51,52], and sleep games are no exception.

Our findings indicate that perceived relevance to sleep health significantly influences the utility value of game features, which in turn leads players to feel more competent in the act of improving sleep health. Features that directly focus on sleep, such as sleep education and sleep rewards in ST, were highly regarded for their relevance and potential to improve sleep. This relevance was considered a major reason why ST could be more effective than the other 2 games. Conversely, features, such as fighting monsters in HSJ, while enjoyable, were not directly linked to sleep health and were consequently rated as the least popular among all HSJ features. Furthermore, several participants expressed concern that features irrelevant to sleep health might distract users from their sleep goals, potentially harming rather than improving sleep. This concern reflects the tension between the health care context and the entertainment nature of games highlighted in previous studies [53]. Addressing this tension requires careful alignment between the serious context and the game features following established design frameworks [41,53]. Indeed, participants made pertinent suggestions on how to better align the game design with sleep health goals, including aligning daily challenges and rewards with the day-night cycle and discouraging oversleeping.

Interestingly, we observed that the perceived utility of sleep game features does not always match their actual utility. Some features, despite having a direct positive impact on nighttime sleep supported by scientific evidence, were not perceived as relevant by many participants. Features such as the daily challenges in HSJ and friendly nudges in ST encourage users to engage in daytime activities conducive to better sleep. These activities represent meaningful behaviors for improving sleep and empower users to achieve their sleep goals. However, not all participants immediately recognized the relevance of these activities and were hesitant to engage with them. For instance, while some participants understood the connection between daytime physical activity and nighttime sleep, others did not. While goal mechanisms have been proven effective in serious games, particularly in educational settings [54], our study shows that it is equally, if not more important, to communicate the rationale behind these goals to users, particularly regarding their logical relevance to improving sleep.

We also found that perceived relevance alone is not sufficient for users to engage with a feature. Despite its perceived relevance, the sleep education feature in ST was the least popular. Instead of presenting educational content in long text, the sleep education feature could be gamified and presented in smaller chunks through gameplay, as exemplified in [55].

#### Design Recommendation 2: Align Game Features With Sleep Health and Help Users Understand the Rationale Behind Design

Educational content of sleep knowledge can be embedded within the game’s narrative and tasks; for example, introducing a character that provides sleep tips as part of the storyline or using interactive elements, such as quizzes, minigames, and challenges that reward users with in-game benefits for learning about sleep health [55].

### Relevance of Sleep Game Features to Players’ Situational Context

#### Overview

The third dimension of relevance for sleep games involves the situational or circumstantial context of users, akin to the notion of situational relevance discussed in prior research [56]. This notion of “life context” has also become an important issue identified in recent behavior change literature [57]. A key finding of this study is that frequent game interactions during the daytime are not always welcomed, as participants’ willingness to engage with those features varied across the day. While sleep games do not necessarily need to be played at night [14], it is important to maximize the situational relevance of daytime interactions to avoid compromising the sense of autonomy.

All 3 games implemented features that address daytime activities. There is a large body of evidence showing that numerous daytime activities can influence nighttime sleep [4,5], making daytime interactions a natural design target for sleep games. However, many participants expressed concerns about being disrupted by notifications irrelevant to the activities at hand, especially during classes or other busy times. Consequently, features likely to generate excessive notifications, such as friendly nudges and sleep education in ST, were rated as the least popular features among the workshop participants. Several participants mentioned that they do not need the sleep game app to dictate their actions, indicating a compromised sense of autonomy due to potentially excessive notifications.

One way to address the potential interruptions caused by excessive notifications is through boundary management [58,59], which ensures that notifications are pushed at appropriate timing and are relevant to the users’ situational context. This can be achieved through direct approaches, such as user customization, or indirect approaches, such as leveraging contextual information and users’ behavior patterns to determine optimal notification timing [60]. For example, many participants were reluctant to engage in sleep games immediately upon waking, as they preferred to prioritize their daily routines over gaming. This observation aligns with previous research indicating that people’s acceptance of notifications varies throughout the day [61] and that they are less likely to attend to notifications in the morning compared with other times of the day [62].

Ensuring the relevance of sleep game features to users’ situational context addresses their psychological needs for autonomy. Besides customizing notifications, participants expressed a desire for autonomy in completing daily challenges to gain a sense of competence. Users prefer daily challenges to be achievable rather than feeling frustrated about not being able to accomplish them. For example, instead of setting a default goal of 10,000 steps, step goals should be customizable because users may have busy schedules, prefer biking over walking, or prefer indoor exercise over outdoor walking on some days. On the basis of these findings, we propose the third design recommendation mentioned subsequently.

#### Design Recommendation 3: Support Boundary Management Through Manual and Automatic Customization

Allow users to choose when and how often they receive notifications. Make it possible for users to opt to receive notifications only in the morning, evening, or at specific times that suit their daily routines. Contextual information from other apps, such as calendar, GPS, and sleep profile, can be used to customize notification settings automatically, avoiding sending notifications during busy periods, meetings, or events. Providing a variety of choices and allowing users to configure a daily challenge list according to their daily schedule is another way to enhance the situational relevance of sleep game features.

### Limitations

This study has several limitations. First, our participants predominantly consist of male international students enrolled in a Japanese university. As a result, the findings may not be readily applicable to other demographic groups. Second, all participants had prior experience playing video games, which could potentially limit the generalizability of our results to individuals who do not engage in gaming. Third, due to the small sample size, we were not able to quantify the associations between player traits and their preferences for sleep game features. We will expand the participant pool to include a more diverse demographic and investigate the preferences of individuals with limited or no prior gaming experience.

### Conclusions

This study examined university students’ preferences for 3 sleep game prototypes and their perceived utility in promoting sleep health. Results showed a notable divide between the popularity and perceived utility of the games. HSJ was favored for enjoyment, while ST was seen as more effective for improving sleep health. This highlights the potential of serious games to enhance sleep hygiene, especially when game features are perceived as relevant. Key factors influencing perceived utility include prior gameplay preferences, perceived relevance to sleep health, and avoiding excessive interactions. Our findings suggest focusing on psychological, logical, and situational relevance in game design. We recommend diversifying game genres, aligning features with sleep health, and managing technology boundaries effectively.

## Abbreviations

HSJ: Hero’s Sleep Journey
KUAS: Kyoto University of Advanced Science
RPG: role-playing game
ST: Sleep Tamagotchi
